# Reported Slowing in Walking and Everyday Activities and Long-Term Mortality in Older Adults: The NEDICES Population-Based Cohort Study

**DOI:** 10.3390/medsci14050524

**Published:** 2026-08-28

**Authors:** Julián Benito-León, Carla María Benito-Rodríguez, Álex Escolà-Gascón, Félix Bermejo-Pareja

**Affiliations:** 1 Department of Neurology, 12 de Octubre University Hospital, Gta. Málaga, 11, Usera, 28041 Madrid, Spain; 2 Group of Neurodegenerative Diseases, Hospital Universitario 12 de Octubre Research Institute (imas12), 28041 Madrid, Spain; fbp.gijon@yahoo.es; 3 Network Center for Biomedical Research in Neurodegenerative Diseases (CIBERNED), 28029 Madrid, Spain; 4 Department of Medicine, Faculty of Medicine, Complutense University of Madrid, 28040 Madrid, Spain; 5 Department of Neurology, Torrejón University Hospital, 28850 Torrejón de Ardoz, Spain; carlaabeenito@gmail.com; 6 Blanquerna Foundation, Ramón Llull University, 08022 Barcelona, Spain; alexeg@blanquerna.url.edu

**Keywords:** reported slowing, walking, everyday activities, subjective motoric complaints, frailty, mortality, aging, cohort study, NEDICES, survival analysis

## Abstract

**Background:** Objective gait speed, self-rated walking pace, and self-reported walking difficulty are consistently associated with mortality. Far less is known about the prognostic meaning of perceiving, or being told, that one has recently become slower in both walking and everyday activities. We examined whether reported slowing was associated with long-term all-cause mortality in older adults. **Methods:** The Neurological Disorders in Central Spain (NEDICES) study is a prospective population-based cohort of 5278 adults aged 65 years or older. At baseline (1994–1995), participants were asked whether they noticed, or had been told, that lately they walked or did things more slowly; the item was available for 3994 participants. Vital status was ascertained through 31 December 2017 by linkage to the Spanish National Population Register. Kaplan–Meier methods and Cox regression were used. The primary model included the Carey-based comorbidity score and adjusted for demographic, movement-disorder, musculoskeletal, and lifestyle covariates. **Results:** Reported slowing was present in 1516 participants (38.0%); 3426 deaths occurred. In the primary complete-case model (*n* = 3858; 3311 deaths), reported slowing was associated with mortality after multivariable adjustment (hazard ratio 1.10, 95% confidence interval 1.02–1.18; *p* = 0.012). The item changed Harrell’s C-index from 0.7046 to 0.7048. The estimate was attenuated after excluding deaths within five years (1.08, 0.99–1.17; *p* = 0.087) and after exploratory adjustment for the Pfeffer Functional Activities Questionnaire score (1.08, 1.00–1.16; *p* = 0.055). **Conclusions:** Reported slowing in walking and everyday activities was associated with a modest increase in mortality after multivariable adjustment. It may serve as a simple indicator of slightly higher mortality risk, although its incremental contribution to individual-level prognostication was limited.

## 1. Introduction

Slowness is one of the most reproducible clinical markers of vulnerability in later life. In a pooled analysis of 34,485 community-dwelling adults, each 0.1 m/s increment in gait speed was associated with a 12% lower hazard of death, and systematic reviews have confirmed a graded relation between usual walking speed and mortality [1,2,3,4]. Prognostic information is not confined to a single baseline walk: improvement in gait speed, longitudinal gait-speed trajectories, long-distance corridor-walk performance, late-midlife walking speed, lower-extremity performance, cardiovascular mortality, and timed hand dexterity have each been related to subsequent survival [5,6,7,8,9,10,11]. Together, these findings suggest that slowness integrates neurological, cardiorespiratory, musculoskeletal, cognitive, and motivational reserve rather than reflecting locomotion alone.

Direct performance testing, however, is not always available. Self-rated walking speed correlates with measured gait speed [12]. Large cohorts have likewise linked slow habitual walking pace with all-cause and cardiovascular mortality [13,14,15,16,17], while reports of limited walking ability or walking difficulty have predicted disability, healthcare use, and death [18,19,20]. A self-reported frailty model incorporating slow walking also predicted disability, falls, and mortality [21]. These observations accord with broader evidence that even a single global self-rating of health can retain prognostic information after multivariable adjustment [22,23].

The evidence closest to perceived recent slowing is more limited but directly relevant. In ambulatory older adults with normal baseline gait speed, five subjective motoric complaints—including reporting that walking had become more difficult than five years earlier—predicted incident objectively slow gait, with adjusted hazard ratios (HRs) ranging from 2.26 to 4.44 [24]. Self-reported motoric cognitive risk syndrome, defined by self-reported slow gait and subjective cognitive decline, was associated with mortality [25], extending conventional motoric cognitive risk findings linking measured slow gait plus cognitive complaints to mortality and dementia [26,27,28]. These constructs show that subjective motor change can precede or accompany adverse outcomes. None of these constructs, however, evaluates the broader report that a person has lately become slower both when walking and when carrying out everyday activities. Direct evidence for that combined, change-based construct remains sparse.

The biological rationale is broad rather than disease-specific. Slowness is a component of physical frailty, and frailty is associated with disability, institutionalization, and death [29,30,31,32]. Physical frailty indicators also predict subsequent activities-of-daily-living disability [33], while slow gait itself has diverse determinants, including weakness, pain, inactivity, obesity, visual impairment, and multimorbidity [34]. A brief report of slowing may therefore aggregate several deficits that are only partly captured by diagnostic labels.

Neurological aging provides a complementary framework. Mild parkinsonian signs in community samples have been associated with mortality, disability, falls, cognitive decline, and mixed cerebrovascular and neurodegenerative pathology [35,36,37,38,39,40,41,42,43]. Self-reported parkinsonian symptoms are common but heterogeneous [44], and the prevalence and functional impact of observed signs vary markedly with definition and age [45]. Thus, a history of slowing may carry neuroepidemiological information without being specific for Parkinson’s disease or parkinsonism.

Against this background, we investigated whether reported recent slowing in walking and everyday activities was associated with long-term all-cause mortality. The analysis was conducted in the population-based Neurological Disorders in Central Spain (NEDICES) cohort, which enrolled 5278 adults aged 65 years or older from three communities in central Spain through a two-phase neurological survey [46,47]. NEDICES has previously supported prospective studies of physical activity and parkinsonism [48] and long-term mortality in Parkinson’s disease [49]. Over more than two decades of follow-up, we assessed the association after adjustment for demographic, movement-disorder, musculoskeletal, lifestyle, and comorbidity measures and examined whether the item improved discrimination beyond these covariates.

## 2. Materials and Methods

### 2.1. Study Design and Population

NEDICES is a prospective, population-based survey of adults aged 65 years or older living in three areas of central Spain: Lista in Madrid, Las Margaritas in Getafe, and the rural county of Arévalo. The baseline survey was conducted in 1994–1995 using a two-phase design: standardized screening by trained lay interviewers followed, when indicated, by neurological assessment and diagnostic adjudication [46,47]. The baseline cohort comprised 5278 screened participants.

**Figure 1:**
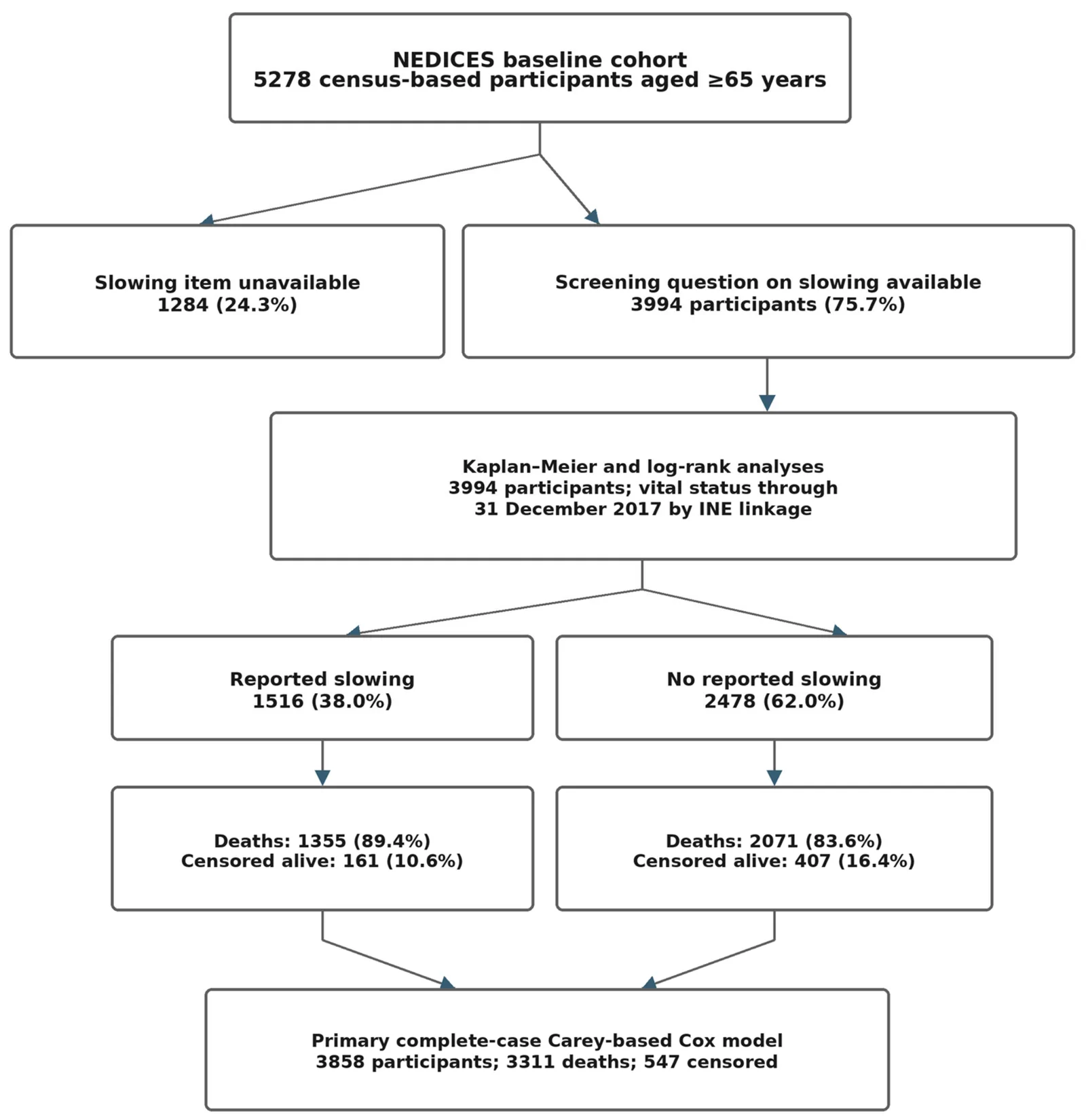
Study flow chart and analytic samples. INE, Instituto Nacional de Estadística.

The present analysis used baseline data (1994–1995) and vital status through 31 December 2017. Information on the exposure of interest was available for 3994 participants (75.7%); 1284 participants (24.3%) lacked this information and were excluded from the primary survival analyses. All 3994 included participants had reliable follow-up time and vital status. Participants with and without information on the slowing item were compared using the available baseline data. The analytic flow is shown in Figure 1.

### 2.2. Exposure: Reported Slowing in Walking and Everyday Activities

The exposure was defined by the third phase-I NEDICES screening question for parkinsonism, using the English wording published in the original study: “Do you notice or have you been told that lately you walk or do things more slowly?” [47]. Participants answering yes were classified as having reported slowing, whereas those answering no were classified as not having reported slowing.

We did not record whether an affirmative response reflected the participant’s own perception or information provided by another person. Separate participant and companion ratings, procedures for resolving disagreement, and item-specific reliability assessments were not available. In addition, “lately” was not defined by a fixed retrospective interval, and the item did not capture the duration, persistence, severity, or cause of the reported slowing. Interviewers were, however, trained to administer the screening questions uniformly [46,47].

When quoting the original NEDICES item, we retain the wording “doing things more slowly.” Elsewhere, we use “everyday activities” descriptively; this should not be interpreted as a formal measure of basic or instrumental activities of daily living.

### 2.3. Mortality Ascertainment

The primary outcome was all-cause mortality. Vital status and dates of death were ascertained through 31 December 2017 by linkage to the Spanish National Population Register (Instituto Nacional de Estadística; INE), the official source used in previous NEDICES mortality analyses [49]. Follow-up time accrued from baseline assessment (1994–1995) to the date of death or to administrative censoring on 31 December 2017 for participants who were alive.

The broad underlying cause-of-death variable was used only for a descriptive comparison among decedents. All-cause mortality was retained as the study outcome, and deaths were not reclassified or censored according to attributed cause in the primary or sensitivity analyses.

### 2.4. Covariates and Functional Status

The primary adjustment set was prespecified on clinical and epidemiological grounds. The model included age, sex, educational level, arterial hypertension, prevalent essential tremor, prevalent Parkinson’s disease, osteoarthritis, osteoporosis, smoking status, alcohol-consumption status, and the Carey-based comorbidity score [50]. Education, smoking, and alcohol were modeled categorically.

A screening question for depressive symptoms was administered at baseline: “Do you suffer from depression?” The yes/no response was used to define depressive symptoms at study entry. The wording was modeled on a previously evaluated single-question screen that identified depression in 85.4% of participants and performed comparably to the 30-item Geriatric Depression Scale [51]. In a NEDICES subsample of 50 participants, the question showed 86% concordance with DSM-IV diagnostic criteria (κ = 0.72; sensitivity, 90.5%; specificity, 82.8%), with similar performance in participants with and without neurological disorders [52].

Comorbidity was summarized using a Carey-based score [50], as previously applied in NEDICES [49]. The score incorporated atrial fibrillation, cancer, chronic obstructive pulmonary disease, depression, dementia, diabetes, treated epilepsy, heart failure, myocardial infarction, psychiatric disorders, renal disease, and stroke, with weighted contributions yielding a total score ranging from 0 to 28. Because depression and dementia are components of this score, neither depressive symptoms nor prevalent dementia was entered separately in the primary model. The score was retained intact rather than modified by removing individual conditions. Arterial hypertension, essential tremor, Parkinson’s disease, osteoarthritis, and osteoporosis were included separately because they were not components of the Carey-based score and were considered directly relevant potential confounders of reported slowing.

In a separate sensitivity analysis, a binary indicator of depressive symptoms and/or antidepressant use was added to the model. Prevalent dementia was additionally examined in exclusion analyses, and another sensitivity model replaced the Carey-based comorbidity score with the available individual morbidity indicators.

Baseline functional status was summarized with an 11-item version of the Pfeffer Functional Activities Questionnaire (FAQ) score [53]. The FAQ assesses ability and dependence in complex instrumental activities; it does not measure movement or task speed. It was included only in an exploratory sensitivity model to examine residual confounding by baseline functional impairment. Because functional limitation may also lie downstream of the processes that produce reported slowing, attenuation in this model cannot distinguish confounding from overadjustment.

### 2.5. Statistical Analysis

Continuous variables were summarized as mean (standard deviation) or median (interquartile range), and categorical variables as counts and percentages. Welch’s *t* test was used for age, Mann–Whitney tests for the Carey-based comorbidity score and Pfeffer FAQ score, and Pearson’s chi-square tests for categorical variables. Standardized mean differences, Cramér’s V/phi, or rank-biserial correlations were reported to place baseline differences in context.

Survival was described with Kaplan–Meier estimates and compared using the log-rank test. Cox proportional-hazards regression with Breslow handling of tied event times estimated HRs and 95% confidence intervals (CIs). Sequential models were fitted on the same primary complete-case cohort: unadjusted; age- and sex-adjusted; additionally adjusted for education; and the fully adjusted Carey-based model.

The proportional-hazards assumption was evaluated using scaled Schoenfeld residual tests for the exposure and each covariate, together with a global test [54]. Because the global test indicated nonproportionality for several adjustment covariates, an extended Cox sensitivity model was fitted with time-varying coefficients for sex, education, osteoporosis, smoking status, alcohol-consumption status, and the Carey-based comorbidity score. These effects were modeled as interactions with log(follow-up time in months + 1), while the coefficient for reported slowing was held constant. Robust standard errors were clustered by participant. Multicollinearity was evaluated using variance inflation factors (VIFs).

Incremental discrimination was explored by comparing the apparent Harrell C-index of the primary model without and with the slowing item [55]. Sensitivity analyses excluded participants with prevalent Parkinson’s disease, prevalent dementia, or either condition; excluded deaths during the first 24 or 60 months; added a binary indicator of depressive symptoms and/or antidepressant use; replaced the Carey-based comorbidity score with the available individual morbidity indicators; added the Pfeffer FAQ score; and used the extended Cox model with time-varying adjustment effects. The cause-of-death table was descriptive and restricted to decedents; it did not estimate cause-specific mortality risk in the analytic cohort or account for competing risks. A two-sided *p* value < 0.05 was considered statistically significant. Analyses were performed using Python 3.13 with statsmodels 0.14.6. Reporting followed STROBE recommendations [56].

### 2.6. Institutional Review and Consent

All procedures involving human participants were approved and overseen by the Clinical Research Ethics Committees of 12 de Octubre University Hospital and La Princesa University Hospital, Madrid, Spain. The study was conducted in accordance with the ethical principles governing human research.

Written informed consent is reported in the dedicated Informed Consent Statement below.

## 3. Results

### 3.1. Availability of the Slowing Item

Of the 5278 baseline participants, 3994 (75.7%) had information on the slowing item, and 1284 (24.3%) did not (Figure 1). Participants with the item were younger than those without it (mean 73.98 vs. 75.34 years; *p* < 0.001). Sex distribution was similar (women 57.1% vs. 59.3%; *p* = 0.164), whereas educational attainment differed (*p* < 0.001) (Table 1).

### 3.2. Baseline Characteristics

Reported slowing was present in 1516 of 3994 participants (38.0%). Compared with those without reported slowing, exposed participants were older and more often women, had lower educational attainment, and had greater comorbidity and functional impairment. The largest baseline effect sizes were observed for age and the Pfeffer FAQ score (both approximately 0.21), education (Cramér’s V 0.14), and osteoporosis (phi 0.12). Several statistically significant differences were small in magnitude (Table 2).

### 3.3. Mortality and Absolute Survival

**Figure 2:**
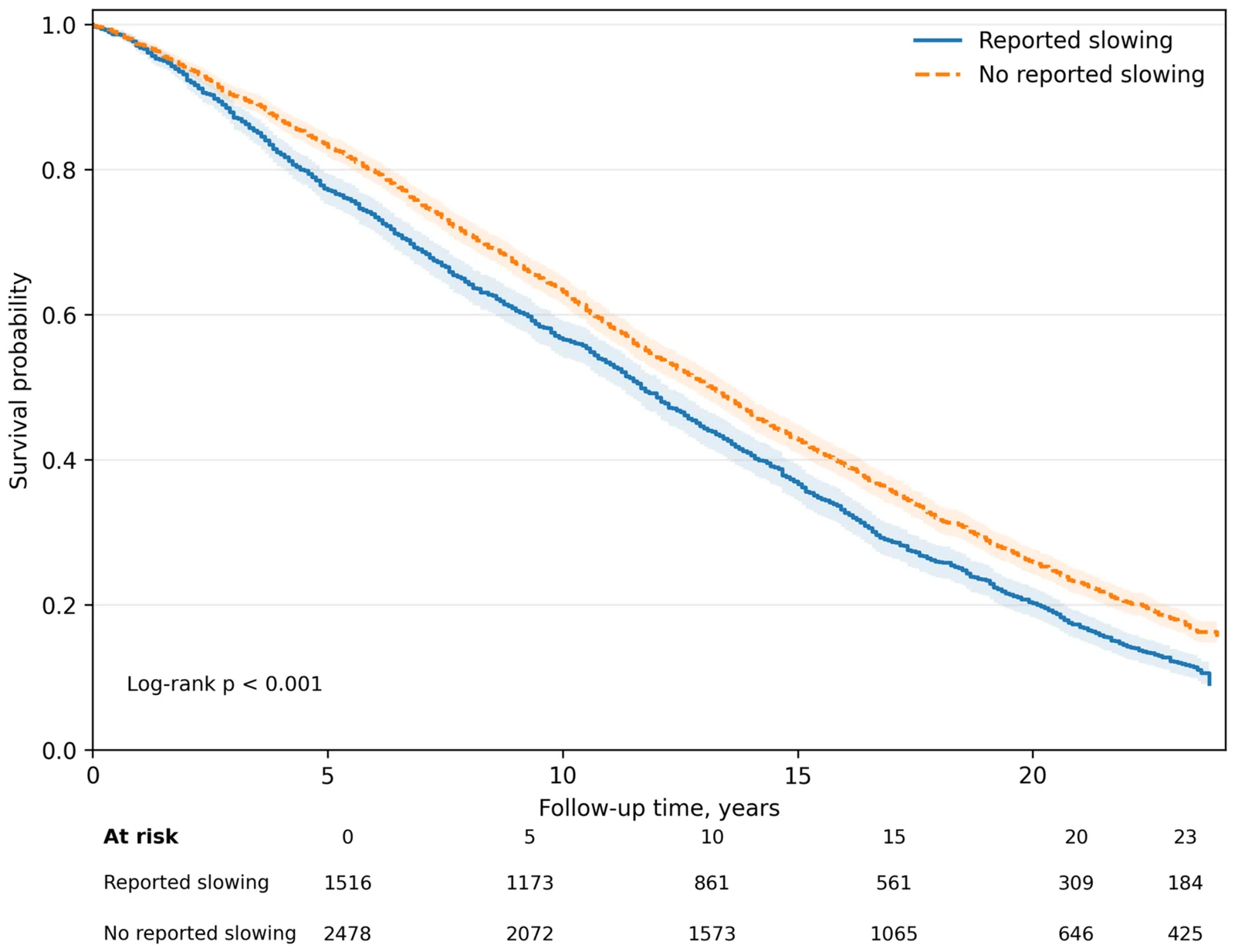
Kaplan–Meier survival curves with numbers at risk according to reported slowing. Shaded areas represent 95% confidence intervals.

Through 31 December 2017, 3426 participants died and 568 were censored alive. Crude mortality was 89.4% among participants with reported slowing and 83.6% among those without it. Kaplan–Meier survival was lower in the reported-slowing group (log-rank chi-square = 32.02; *p* < 0.001; Figure 2). Estimated survival was 77.2% versus 83.1% at 5 years, 56.5% versus 63.2% at 10 years, and 36.7% versus 42.8% at 15 years (Table 3).

### 3.4. Sequential Cox Models and Incremental Discrimination

The HR attenuated from 1.22 in the unadjusted model to 1.10 after full adjustment, indicating that a substantial proportion of the crude association was explained by measured group differences. In the primary complete-case model (*n* = 3858; 3311 deaths), reported slowing remained associated with mortality after multivariable adjustment (HR 1.10, 95% CI 1.02–1.18; *p* = 0.012). However, adding the item changed the apparent Harrell C-index only from 0.7046 to 0.7048 (Δ = 0.0002), indicating no material improvement in individual-level discrimination (Table 4).

### 3.5. Sensitivity and Alternative-Specification Analyses

**Figure 3:**
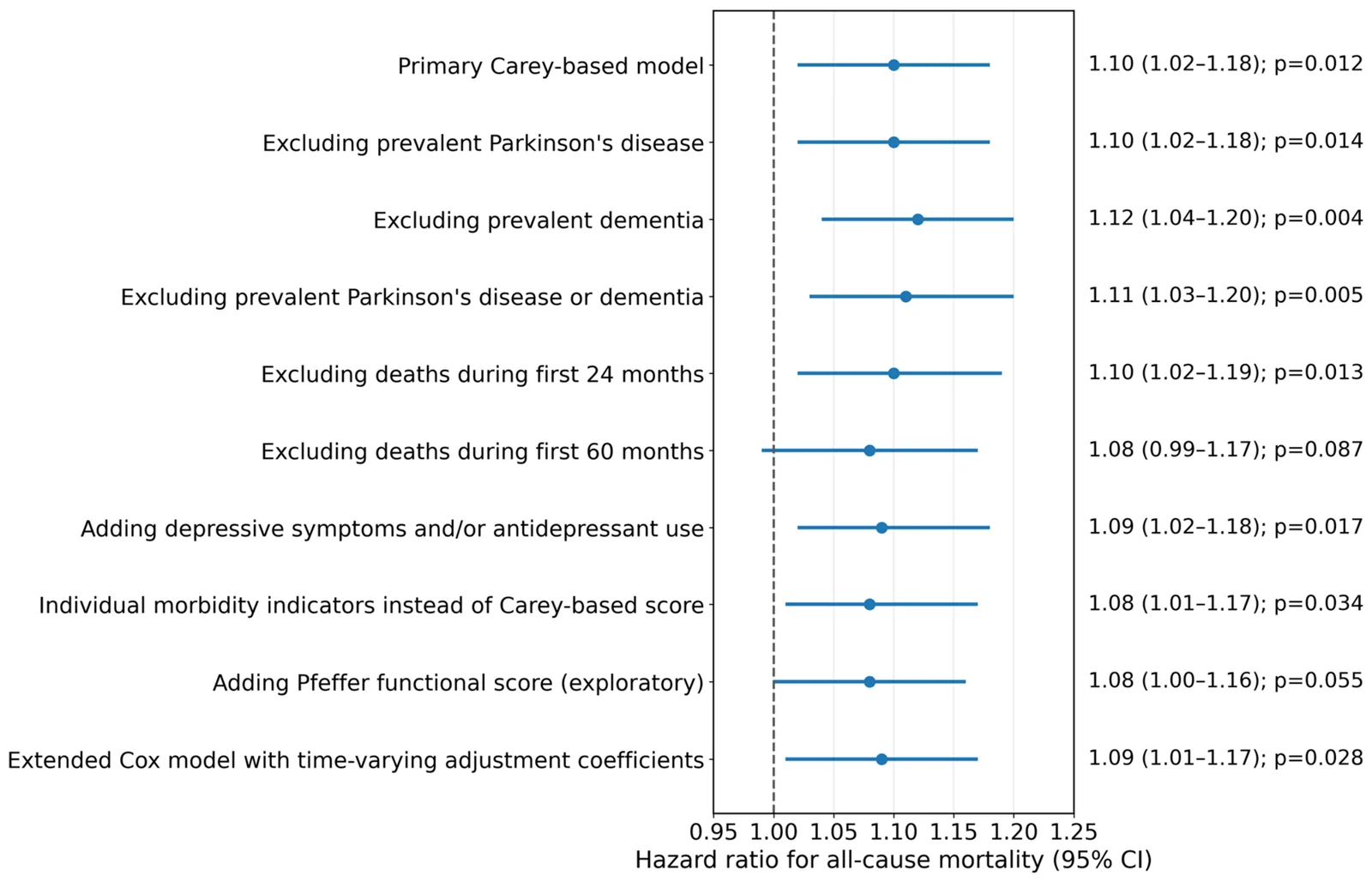
Multivariable association between reported slowing and all-cause mortality across sensitivity and alternative-specification analyses. Blue points represent adjusted hazard ratios, horizontal lines represent 95% confidence intervals, and the vertical dashed line denotes the null value (HR = 1.00). HR, hazard ratio; CI, confidence interval.

The effect estimate changed little after excluding participants with prevalent Parkinson’s disease, prevalent dementia, either condition, or deaths occurring within the first 24 months of follow-up. After excluding deaths occurring within the first 60 months, the association was attenuated, and the 95% confidence interval included the null (HR 1.08, 95% CI 0.99–1.17; *p* = 0.087). Adding a binary indicator of depressive symptoms and/or antidepressant use to the Carey-based model or replacing the Carey-based index with the available individual morbidity indicators yielded similarly modest estimates. Exploratory adjustment for the Pfeffer FAQ score further attenuated the association (HR 1.08, 95% CI 1.00–1.16; *p* = 0.055). The extended Cox model allowing time-varying coefficients for all adjustment factors with evidence of nonproportionality yielded a slightly attenuated estimate for reported slowing (HR 1.09, 95% CI 1.01–1.17; *p* = 0.028) (Table 5; Figure 3).

### 3.6. Proportional-Hazards and Multicollinearity Diagnostics

**Figure 4:**
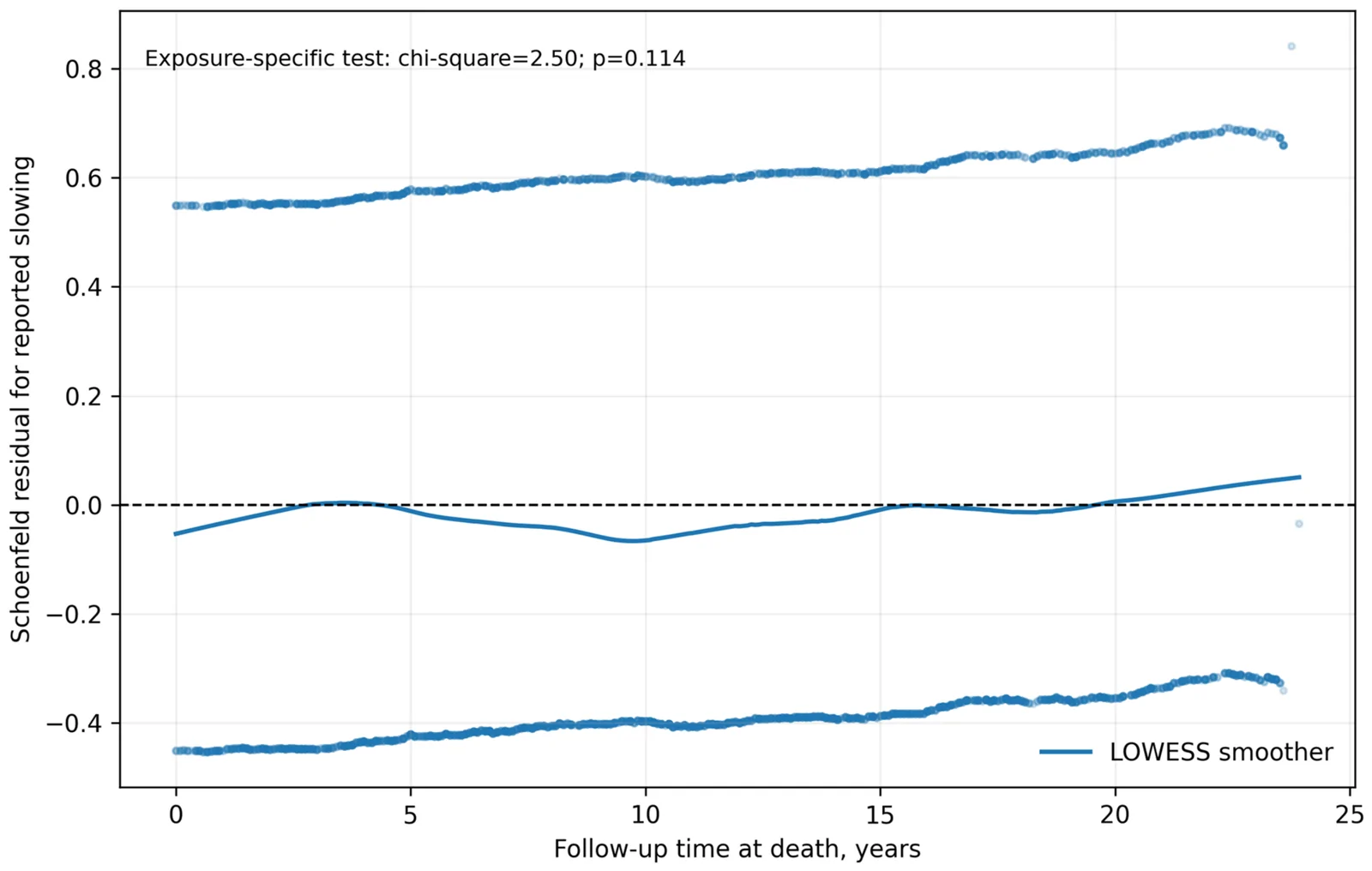
Scaled Schoenfeld residuals for the reported-slowing exposure against follow-up time. Blue points represent the scaled Schoenfeld residuals, the solid blue line is the LOWESS smoother, and the horizontal dashed line denotes zero. The formal exposure-specific test is reported in Table 6.

The exposure-specific scaled Schoenfeld residual test did not indicate departure from proportional hazards (chi-square = 2.50; *p* = 0.114). The global test was significant (chi-square = 105.85; 16 degrees of freedom; *p* < 0.001), with evidence of time dependence for sex, education, osteoporosis, smoking status, former alcohol use, and the Carey-based comorbidity score. In the extended Cox model allowing these adjustment effects to vary with log(follow-up time in months + 1), the association between reported slowing and mortality was similar in magnitude (HR 1.09, 95% CI 1.01–1.17; *p* = 0.028). The maximum non-intercept VIF was 2.43, providing no evidence of problematic multicollinearity (Table 6; Figure 4).

### 3.7. Descriptive Cause-of-Death Distribution

Among the 3426 decedents, 3392 (99.0%) had a coded broad cause of death; 34 had an unknown or uncoded cause and were excluded. The proportional distribution of causes of death did not differ significantly between exposure groups (Pearson chi-square = 6.23; 5 degrees of freedom; *p* = 0.284; Table 7). Because the analysis was restricted to decedents, it did not estimate cause-specific mortality risk in the 3994-participant analytic cohort and should not be interpreted etiologically.

## 4. Discussion

In this population-based cohort of older adults, reported slowing in walking and everyday activities was associated with higher all-cause mortality over long-term follow-up. The association was attenuated after multivariable adjustment, with the hazard ratio decreasing from 1.22 to 1.10, but remained statistically significant. Adding the slowing item produced only a minimal change in Harrell’s C-index (0.7046 to 0.7048), indicating limited incremental discrimination beyond the covariates already included in the model.

The result lies between two well-developed bodies of evidence. Objective gait speed and its change over time predict mortality across diverse cohorts [1,2,3,4,5,6,7,8,9,10,11], while self-rated pace, walking ability, and walking difficulty retain prognostic information without formal performance testing [12,13,14,15,16,17,18,19,20,21,22,23]. Subjective motoric complaints can precede objectively slow gait [24], and both self-reported and conventional motoric cognitive risk phenotypes are associated with mortality or dementia [25,26,27,28]. The present exposure is nevertheless different: it asks about a perceived recent change and combines walking with a broad sense of becoming slower in everyday activity. This distinction—change rather than habitual pace and a wider domain than walking alone—defines the study’s specific contribution.

The phrase “doing things more slowly” is broad and does not specify a particular activity or correspond to a formal measure of basic or instrumental activities of daily living. An affirmative response may nevertheless reflect functional vulnerability, frailty, pain, fatigue, mood symptoms, musculoskeletal disease, medication effects, or systemic illness [29,30,31,32,33,34]. The Pfeffer FAQ addresses ability and dependence in complex instrumental activities, not speed of performance [53]. Its inclusion in an exploratory model attenuated the hazard ratio to 1.08. That attenuation is compatible with confounding by baseline functional impairment, but it could also reflect overadjustment if functional loss lies on the pathway from underlying illness to reported slowing. The Pfeffer model therefore informs interpretation without replacing the prespecified primary model.

The original survey context also matters. The item was one component of a two-phase parkinsonism screen [46,47], and late-life parkinsonian signs are associated with disability, cognitive decline, mixed pathology, and mortality [35,36,37,38,39,40,41,42,43,44,45]. A positive response did not, however, establish a diagnosis; screen-positive participants underwent neurological assessment in the second phase [46,47]. Consistent with that design, excluding prevalent Parkinson’s disease or dementia did not remove the association, but neither does multivariable adjustment establish neurological specificity. The most defensible interpretation is a broad, nonspecific marker associated with modestly elevated mortality risk, not a proxy diagnosis.

The alternative model specifications produced estimates of similar, modest magnitude. Adding a binary indicator of depressive symptoms and/or antidepressant use, or replacing the Carey-based score with the available individual morbidity indicators, did not materially alter the estimate. Thus, the association did not depend on one particular covariate formulation, although its adjusted magnitude remained modest.

The lag analyses narrow the temporal interpretation. Excluding deaths during the first 24 months had little effect, whereas excluding deaths within five years reduced the estimate to 1.08 and the confidence interval included the null. Because a lag analysis removes deaths most plausibly related to illness already advanced at baseline, this attenuation raises the possibility of reverse causation: the question may identify people already entering a period of decline more than it identifies a stable long-term trait. It does not negate the primary result, but it limits causal claims and suggests that the signal may be most relevant to relatively proximate vulnerability.

The exposure-specific Schoenfeld test did not indicate nonproportionality, whereas the global test identified time dependence in several adjustment covariates. Allowing the coefficients for all affected adjustment factors to vary with follow-up time slightly attenuated the estimate for reported slowing but did not materially change its magnitude. This reduces concern that the exposure estimate arose solely from misspecification of the adjustment covariates, although it does not remove the study’s other limitations. The negligible change in C-index likewise indicates limited incremental individual-level discrimination.

The cause-of-death findings require a similarly bounded reading. The proportional distribution of broad causes among decedents was comparable between exposure groups, but a decedent-only comparison does not estimate cause-specific mortality risk and cannot identify a biological pathway. Cause-specific Cox or competing-risk models would be required to assess whether associations differ across causes of death. In addition, all-cause mortality necessarily includes deaths that may be unrelated to chronic age-related deterioration, such as accidents or some acute illnesses; these events may have influenced the observed association.

Selection and measurement limitations remain important. Nearly one quarter of the baseline cohort lacked information on the slowing item. Although baseline characteristics could be compared between participants with and without exposure information, the direction and magnitude of any resulting selection bias cannot be determined. The study did not distinguish between the participant’s own perception and information communicated by another person, nor did it record whether a companion was present or how disagreements, if any, were resolved. The term “lately” was not tied to a predefined time interval, and the item did not distinguish transient from persistent slowing or capture its severity, laterality, associated falls or freezing, pain, fatigue, or objective gait speed. Item-specific reliability data were also unavailable.

The long follow-up is both a strength and a constraint. Population-based sampling, standardized neurological ascertainment, a large number of deaths, and national registry linkage support internal validity, but a single baseline report cannot represent changes occurring over more than two decades. Incident Parkinson’s disease, dementia, stroke, frailty, and other conditions may have mediated or otherwise explained part of the association. Repeated assessment and paired objective gait measures would be needed to distinguish persistent perceived slowing from transient symptoms and evolving disease.

The practical implication is deliberately limited. A report of recent slowing should not be treated as a deterministic prognostic test, as evidence that walking faster would reduce mortality, or as a substitute for neurological and functional assessment. It may, however, act as a concise clinical signal that prompts attention to mobility, cognition, mood, pain, medication, musculoskeletal disease, frailty, and systemic health. External validation using the same wording, explicit separation of participant and informant reports, and repeated objective and subjective measurements would clarify whether this signal is reproducible and when it is most informative.

## 5. Conclusions

In NEDICES, reported slowing in walking and everyday activities was associated with a small increase in all-cause mortality after multivariable adjustment. The association weakened after a five-year lag and exploratory adjustment for functional status, while the item added virtually no discrimination beyond the covariates already included in the model. These findings indicate that reported slowing may identify a group with slightly higher mortality risk, although its contribution to individual-level prognostication was limited.

## Figures and Tables

**Table 1:** Baseline comparison of participants with and without information on the slowing item.

Characteristic	Item Available (*n* = 3994)	Item Unavailable (*n* = 1284)	*p* Value	Effect Size
Age, years, mean (SD)	73.98 (6.72)	75.34 (7.62)	<0.001	−0.19
Men, *n* (%)	1715 (42.9)	523 (40.7)	0.164	0.02
Women, *n* (%)	2279 (57.1)	761 (59.3)		
Education available, *n*	3994	1237		
Illiterate, *n* (%)	552 (13.8)	159 (12.9)	<0.001	0.09
Can read and write, *n* (%)	1652 (41.4)	440 (35.6)		
Primary studies, *n* (%)	1222 (30.6)	498 (40.3)		
Secondary or higher, *n* (%)	568 (14.2)	140 (11.3)		

Effect sizes are reported as the standardized mean difference for age, phi for sex, and Cramér’s V for education. Percentages for education are based on participants with educational data (*n* = 5231).

**Table 2:** Baseline characteristics according to reported slowing.

Characteristic	Reported Slowing (*n* = 1516)	No Reported Slowing (*n* = 2478)	*p* Value	Effect Size
Age, years, mean (SD)	74.8 (6.8)	73.5 (6.6)	<0.001	0.21
Female sex, *n* (%)	934 (61.6)	1345 (54.3)	<0.001	0.07
Education: illiterate, *n* (%)	230 (15.2)	322 (13.0)	<0.001	0.14
Education: can read/write, *n* (%)	604 (39.8)	1048 (42.3)		
Education: primary studies, *n* (%)	545 (35.9)	677 (27.3)		
Education: secondary/higher, *n* (%)	137 (9.0)	431 (17.4)		
Carey-based comorbidity score, median (IQR)	1 (0–2)	0 (0–2)	<0.001	0.09
Pfeffer FAQ score, median (IQR)	1 (0–4)	0 (0–1)	<0.001	0.21
Arterial hypertension, *n*/*N* (%)	823/1513 (54.4)	1222/2473 (49.4)	0.002	0.05
Prevalent essential tremor, *n*/*N* (%)	115/1516 (7.6)	104/2478 (4.2)	<0.001	0.07
Prevalent Parkinson’s disease, *n*/*N* (%)	52/1516 (3.4)	16/2478 (0.6)	<0.001	0.10
Osteoarthritis, *n*/*N* (%)	972/1496 (65.0)	1448/2454 (59.0)	<0.001	0.06
Osteoporosis, *n*/*N* (%)	317/1483 (21.4)	297/2423 (12.3)	<0.001	0.12
Depressive symptoms and/or antidepressant use, *n*/*N* (%)	466/1503 (31.0)	573/2465 (23.2)	<0.001	0.09
Prevalent dementia, *n*/*N* (%)	100/1516 (6.6)	83/2478 (3.3)	<0.001	0.08
Stroke, *n*/*N* (%)	111/1516 (7.3)	103/2478 (4.2)	<0.001	0.07
Chronic obstructive pulmonary disease, *n*/*N* (%)	274/1504 (18.2)	370/2456 (15.1)	0.009	0.04
Cancer, *n*/*N* (%)	103/1508 (6.8)	152/2460 (6.2)	0.417	0.01
Diabetes, *n*/*N* (%)	268/1506 (17.8)	417/2457 (17.0)	0.506	0.01
Heart disease, *n*/*N* (%)	160/1511 (10.6)	228/2473 (9.2)	0.157	0.02
Antiseizure treatment, *n*/*N* (%)	25/1516 (1.6)	26/2478 (1.0)	0.101	0.03
Alcohol consumption: current, *n*/*N* (%)	398/1515 (26.3)	933/2473 (37.7)	<0.001	0.13
Alcohol consumption: former, *n*/*N* (%)	309/1515 (20.4)	526/2473 (21.3)		
Alcohol consumption: never, *n*/*N* (%)	808/1515 (53.3)	1014/2473 (41.0)		
Smoking status: current, *n*/*N* (%)	146/1514 (9.6)	339/2475 (13.7)	<0.001	0.11
Smoking status: former, *n*/*N* (%)	344/1514 (22.7)	730/2475 (29.5)		
Smoking status: never, *n*/*N* (%)	1024/1514 (67.6)	1406/2475 (56.8)		

IQR, interquartile range; SD, standard deviation; FAQ, Functional Activities Questionnaire; *n*/*N*, number with the characteristic/number with available data. Effect sizes are standardized mean differences for age, Cramér’s V/phi for categorical variables, and rank-biserial correlations for Carey-based comorbidity and Pfeffer FAQ scores. The *p* value for education, alcohol, and smoking refers to the overall distribution. The binary indicator of depressive symptoms and/or antidepressant use was coded positive when the baseline question “Do you suffer from depression?” was answered affirmatively and/or antidepressant use was recorded. Missing or unknown values were excluded; denominators are shown when they differed from group totals.

**Table 3:** Mortality and Kaplan–Meier survival according to reported slowing.

Measure	Reported Slowing	No Reported Slowing	Overall
Deaths, *n* (%)	1355 (89.4)	2071 (83.6)	3426 (85.8)
Censored alive, *n* (%)	161 (10.6)	407 (16.4)	568 (14.2)
Mean observed time to death/censoring, months	145.4	160.3	154.7
Median observed time to death/censoring, months	140	158	151
5-year survival (95% CI)	77.2% (75.0–79.2)	83.1% (81.6–84.5)	—
10-year survival (95% CI)	56.5% (54.0–59.0)	63.2% (61.2–65.0)	—
15-year survival (95% CI)	36.7% (34.3–39.1)	42.8% (40.8–44.7)	—

CI, confidence interval; —, not applicable. Participants alive on 31 December 2017 were right-censored at that date. Log-rank chi-square = 32.02; *p* < 0.001.

**Table 4:** Sequential Cox models for all-cause mortality.

Model	Adjustment	Participants/Deaths	HR (95% CI)	*p* Value	Harrell C-Index
1	Unadjusted	3858/3311	1.22 (1.14–1.31)	<0.001	—
2	Age and sex	3858/3311	1.16 (1.08–1.25)	<0.001	—
3	Age, sex, and education	3858/3311	1.16 (1.08–1.25)	<0.001	—
4	Primary Carey-based model	3858/3311	1.10 (1.02–1.18)	0.012	0.7048
Model 4 without the slowing item	Same adjustment set, excluding exposure	3858/3311	—	—	0.7046

HR, hazard ratio; CI, confidence interval; —, not applicable or not calculated. All sequential models used the same primary complete-case cohort. The primary model included age, sex, education, arterial hypertension, prevalent essential tremor, prevalent Parkinson’s disease, osteoarthritis, osteoporosis, smoking, alcohol, and the Carey-based comorbidity score.

**Table 5:** Sensitivity and alternative-specification analyses.

Analysis	Participants/Deaths	HR (95% CI)	*p* Value
Primary Carey-based model	3858/3311	1.10 (1.02–1.18)	0.012
Excluding prevalent Parkinson’s disease	3792/3246	1.10 (1.02–1.18)	0.014
Excluding prevalent dementia	3684/3138	1.12 (1.04–1.20)	0.004
Excluding prevalent Parkinson’s disease or dementia	3629/3084	1.11 (1.03–1.20)	0.005
Excluding deaths during first 24 months	3607/3060	1.10 (1.02–1.19)	0.013
Excluding deaths during first 60 months	3126/2579	1.08 (0.99–1.17)	0.087
Adding depressive symptoms and/or antidepressant use	3841/3296	1.09 (1.02–1.18)	0.017
Available individual morbidity indicators instead of a Carey-based comorbidity score	3767/3230	1.08 (1.01–1.17)	0.034
Adding the Pfeffer Functional Activities Questionnaire score (exploratory)	3849/3302	1.08 (1.00–1.16)	0.055
Extended Cox model with time-varying adjustment coefficients	3858/3311	1.09 (1.01–1.17)	0.028

HR, hazard ratio; CI, confidence interval. The Pfeffer model was exploratory and was not used as the primary specification. The extended Cox model allowed coefficients for sex, education, osteoporosis, smoking status, alcohol-consumption status, and the Carey-based comorbidity score to vary with log(follow-up time in months + 1); robust standard errors were clustered by participant.

**Table 6:** Scaled Schoenfeld residual tests and variance inflation factors for the primary model.

Covariate	PH Chi-Square	*p* Value	VIF
Reported slowing	2.50	0.114	1.08
Age	0.93	0.334	1.07
Female sex	17.74	<0.001	2.38
Education: read/write	0.04	0.837	2.43
Education: primary	4.30	0.038	2.33
Education: secondary/higher	24.65	<0.001	1.85
Arterial hypertension	0.47	0.492	1.04
Prevalent essential tremor	1.52	0.218	1.01
Prevalent Parkinson’s disease	0.01	0.921	1.02
Osteoarthritis	3.59	0.058	1.13
Osteoporosis	4.01	0.045	1.11
Current smoking	6.54	0.011	1.62
Former smoking	10.66	0.001	2.15
Current alcohol use	0.00	0.980	1.60
Former alcohol use	13.31	<0.001	1.39
Carey-based comorbidity score	46.75	<0.001	1.08

PH, proportional hazards; VIF, variance inflation factor. Global PH test: chi-square = 105.85, 16 df, *p* < 0.001. Variables with evidence of nonproportionality were sex, education, osteoporosis, smoking status, former alcohol use, and the Carey-based comorbidity score; these effects were modeled with time-varying coefficients in the extended Cox sensitivity model. Maximum non-intercept VIF = 2.43.

**Table 7:** Broad cause-of-death distribution among decedents with a coded cause.

Cause of Death	Reported Slowing (*n* = 1344)	No Reported Slowing (*n* = 2048)	Overall (*n* = 3392)
Dementia	97 (7.2)	167 (8.2)	264 (7.8)
Cancer	267 (19.9)	446 (21.8)	713 (21.0)
Cardiocirculatory diseases	373 (27.8)	556 (27.1)	929 (27.4)
Cerebrovascular diseases	126 (9.4)	152 (7.4)	278 (8.2)
Respiratory diseases	212 (15.8)	320 (15.6)	532 (15.7)
Other causes	269 (20.0)	407 (19.9)	676 (19.9)
Total known broad cause	1344 (100.0)	2048 (100.0)	3392 (100.0)

Values are *n* (%) with column percentages among decedents with a coded broad cause. Unknown/uncoded causes (*n* = 34) were excluded. Pearson chi-square = 6.23; 5 df; *p* = 0.284. This is a descriptive decedent-only analysis, not an estimate of cause-specific mortality risk.

## Data Availability

The original contributions presented in this study are included in the article. Further inquiries can be directed to the corresponding author.
